# Applying the theory of planned behaviour to multiple sclerosis patients’ decisions on disease modifying therapy – questionnaire concept and validation

**DOI:** 10.1186/1472-6947-12-60

**Published:** 2012-07-02

**Authors:** Jürgen Kasper, Sascha Köpke, Korbinian Fischer, Nina Schäffler, Imke Backhus, Alessandra Solari, Christoph Heesen

**Affiliations:** 1 Institute of Neuroimmunology and Clinical MS-Research (inims) and Department of Neurology, University Medical Center, Hamburg, Germany; 2 Department of Primary Medical Care, University Medical Center, Hamburg, Germany; 3 Faculty of Mathematics, Informatics, Unit of Health Sciences and Education, University of Hamburg, Hamburg, Germany; 4 Institute of Social Medicine, Nursing Research Group, University of Lübeck, Lübeck, Germany; 5 Neuroepidemiology Unit, Foundation IRCCS Neurological Institute C. Besta, Milan, Italy

## Abstract

**Background:**

Patients making important medical decisions need to evaluate complex information in the light of their own beliefs, attitudes and priorities. The process can be considered in terms of the theory of planned behaviour.

Decision support technologies aim at helping patients making informed treatment choices. Instruments assessing informed choices need to include risk knowledge, attitude (towards therapy) and actual uptake. However, mechanisms by which decision support achieves its goals are poorly understood.

Our aim was therefore to develop and validate an instrument modeling the process of multiple sclerosis (MS) patients’ decision making about whether to undergo disease modifying (immuno-)therapies (DMT).

**Methods:**

We constructed a 30-item patient administered questionnaire to access the elaboration of decisions about DMT in MS according to the theory of planned behaviour. MS-patients’ belief composites regarding immunotherapy were classified according to the domains “attitude”, “subjective social norm” and “control beliefs” and within each domain to either “expectations” or “values” yielding 6 sub-domains. A randomized controlled trial (n = 192) evaluating an evidence based educational intervention tested the instrument’s predictive power regarding intention to use immunotherapy and its sensitivity to the intervention.

**Results:**

The psychometric properties of the questionnaire were satisfactory (mean item difficulty 62, mean SD 0.9, range 0–3). Responses explain up to 68% of the variability in the intention to use DMT was explained by up to 68% in the total sample. Four weeks after an educational intervention, predictive power was higher in the intervention (IG) compared to the control group (CG) (intention estimate: CG 56% / IG 69%, p = .179; three domains CG 56% / IG 74%, p = .047; six sub-domains CG 64% / IG 78%, p = .073). The IG held more critical beliefs towards immunotherapy (p = .002) and were less willing to comply with social norm (p = .012).

**Conclusions:**

The questionnaire seems to provide a valid way of explaining patients’ inherent decision processes and to be sensitive towards varying levels of elaboration. Similar tools based on the theory of planned behaviour could be applied to other decision making scenarios.

## Background

Quality of decisions in health care is increasingly viewed as sensitive to individual factors such as preferences and beliefs which on the level of groups and of populations are very difficult to determine [1,2]. Since concrete choices reveal little about decision quality, the process of making a decision might be a better quality indicator [3-5]. This process is predominantly performed internally and includes negotiating information about possible benefit and side-effects with preferences, values and risk attitudes [6]. As therefore, quality refers to both highly individual and intrapersonal criteria, the challenge of assessment is obvious. Accordingly, an ideal decision making process would mean implying cognitive and emotional appraisal of relevant information with anticipated related consequences [7,8]. The latter has been defined as “informed decision” which can be considered as meeting two conditions: first, the patient should have processed the relevant risk knowledge and second, the choice should reflect the patient’s values [6]. Based on this definition, the Multimodal Measure of Informed Choice (MMIC) [3] has been used as an endpoint for studies on decision support. The MMIC comprises three dichotomous measures, knowledge (good, poor), value (positive, negative) and choice (uptake or non-uptake of the intervention under consideration) leading to eight types of choices Only two of these indicate informed choice: either an informed patient’s values are in favour of the intervention which is applied by the patient, or an informed patient’s values are against the intervention which is rejected. Marteau et al. define value as a basic attitude, which referring to Ajzen is a person’s overall evaluation of the behaviour in question [9]. However, the process of decisions and the mechanisms or moderators by which attitude impacts on behaviour cannot be derived from MMIC, which merely assesses the result of this process. Other intrapersonal characteristics also influence the decision making process [10]. Furthermore, different internal considerations – such as beliefs about the consequences of an action or the individual’s own ability to control the situation - can lead to the same choices. Frequencies of informed choice decisions measured using Marteau’s method do not explain how they were achieved.

Elaboration of health related decisions i.e. the motivational process an individual goes through when anticipating an action - the so called action regulation - are seen as internal cognitive processes by a group of theories such as the health belief model, the social–cognitive theory, the theory of reasoned action, the theory of planned behaviour (TPB) and the protection motivation theory [11]. These theories share the assumption that each behaviour is predominantly a function of attitudes and beliefs, as well as expectations of future events and outcomes. Facing various alternatives, individuals will choose the action most likely leading to positive outcomes. Among these theories, the TPB [9] is one of the best proven to explain a specific health behaviour by a set of domains in a wide number of research areas [12-14]. Prediction of self-reported behaviour is superior to observed behaviour. However, according to the literature TPB is capable of explaining 20% of the variance in prospective measures of actual behaviour (and 27 to 39% of intention) [15-18].

The TBP postulates three conceptually independent domains determining an intention to perform a specific behaviour: "attitude" refers to the degree to which a person has a favourable or unfavourable appraisal of the behaviour in question; "social norm" refers to the perceived social pressure to perform/not perform the behaviour; "perceived behaviour control" refers to the perceived ease or difficulty of performing the behaviour and is assumed to reflect past experiences as well as anticipated impediments and obstacles. The theory is based on the expectancy-value model, assuming that overall evaluation of decisional options often contains two separable sub-domains: an expected outcome and a given value [7]. For example, the subjective value of a given outcome such as reduction of the number of relapses in multiple sclerosis as the benefit from disease modifying treatment (DMT) affects the attitude in direct proportion to the strength of the belief regarding occurrence of this outcome. A patient might be convinced of the efficacy of DMT regarding reduction of relapse rates but on the other hand might not prioritize this goal against others leading to low impact of this belief on this patient’s attitude. As a general rule, the more favourable the attitude and subjective norm with respect to a specific behaviour and the greater the perceived behavioural control the stronger is an individual’s intention to perform the behaviour under consideration. The relative weight by which the three domains (attitude, subjective social norm, perceived behavioural control) impact on the intention is expected to vary across different behaviours and contexts.

Since, although not conclusive, the evidence for the TPB’s validity to a broad variety of behavioural decisions is promising, we choose the model to validate theoretical assumptions underpinning our developments of decision support strategies for patients with multiple sclerosis [19-21].

Multiple sclerosis (MS) is a chronic-progressive disease of young adults with a presumed autoimmune aetiology [22]. People affected by the diseases have to deal with pronounced uncertainty regarding prognosis and also regarding effectiveness of available treatments. Hitherto, no curative treatment exists. However, appearance of new relapses, new lesions on magnetic resonance imaging and progression of disability can be slowed down by DMT but at the risk of side effects that can be long lasting and sometime serious [19]. Therefore, decisions about disease modifying treatments (DMT) are highly sensitive to patient preferences [23]. In addition, MS patients claim active roles in these decision making processes which seem even higher than in other diseases [24,25].

This paper describes development and validation of the questionnaire “Planned behaviour in MS” (PBMS), a patient-reported instrument assessing the process of decision making about DMT. By improving understanding of processes underlying patients’ choices on DMT as well as improving understanding of mechanisms mediating effects of decision support strategies, we aim at tailoring decision aids and other support strategies more to patients’ needs.

## Methods

### Ethics statement

The study protocol was approved by the ethics committee of the Hamburg Chamber of Physicians (ref: PV3164) on 12th March 2009 All participants gave written informed consent for recording, analysis and publication of their data collected within this study.

### Questionnaire concept and development

**Figure 1:**
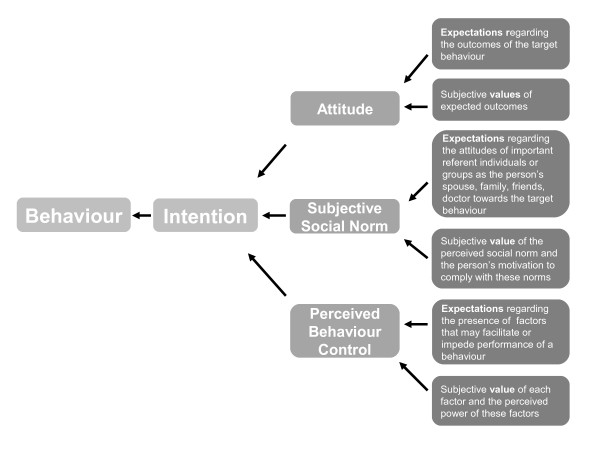
**Illustration of theory of planned behaviour, I Ajzen **[9].

The development of the PBMS - a questionnaire intended to predict or explain patient choices to use or not to use DMT - was based on the instructions given by Ajzen [26]. We focused on assessing all relevant cognitive and emotional issues (belief composites) presumably processed by MS-patients considering a decision about DMT. The original pool of about 120 belief composites was generated by extracting salient statements from 80 videos of physician-patient consultations on DMT decisions [19]. Statements identified as relevant were classified according to their underlying constructs in terms of the TPB framework. They were allocated to one of the three domains (attitude, subjective social norm, perceived behaviour control) and then within each domain to one of two sub-domains (expectations and values) (Figure 1). For example, a recently diagnosed MS patient might be convinced (attitude) that early MS treatment in general is beneficial (expectation) while assigning higher value to potential side effects (value). Statements were identified for all components of the TPB. However, few statements related to control beliefs and in particular assumed power of control. This pool of belief composites and their allocation were discussed and supplemented by an expert panel of neurologists (CH, NS), health scientists (SK, IB) and a psychologist (JK) all experienced in MS treatment decision-making. During this process, classification of the statements was checked and about 20 new statements were added from physicians’ clinical practice. In the next step, based on the underlying concept items were created to exhaustively cover the TPB framework for the scenario of decision making on DMTs. With regard to uniqueness, similarity and disjunctiveness, 38 items were selected and piloted with 10 MS patients in the MS outpatient clinic to check comprehensibility and relevance.

The final questionnaire consisted of 30 items including three cumulatively constructed scales for the TPB domains and six sub-domains accordingly: domain 1: Attitude, 12 items ((1a) 5 items assessing *“Expectations regarding outcomes of DMT”* and (1b) 7 items assessing “*Subjective values of expected outcomes*”), domain 2: Subjective social norms, 10 items ((2a) 4 items assessing “*Expectations regarding attitudes of important reference individuals or groups*” and (2b) 6 items assessing “*Subjective value of perceived social norm and the person’s motivation to comply with these norms*”), and domain 3: Control beliefs, 8 items ((3a) 4 items assessing “*Expectations regarding the presence of factors that may facilitate or impede successful DMT*” and (3b) 4 items assessing “*Subjective values and perceived power of these factors*”) (Additional file 1). Items were formulated as statements to be answered by specifying one’s extent of agreement on a four-point Likert scale (I disagree/I somewhat disagree/I somewhat agree/I agree). Within each scale the numbers of items in favour of and against DMT were balanced. Items from the three scales were mixed in the questionnaire (Additional file 1).

### Pre-testing

Psychometric properties of the PBMS, in particular item difficulties and variance, were investigated in a sample of 50 MS patients with relapsing-remitting disease from the MS out patient clinic at the University Medical Centre. Of these, 34 participated in the pilot phase of a 4-hour patient education programme about diagnosis, prognosis and early treatment of multiple sclerosis (PEPADIP, ISRCTN12440282) [27]. To allow for exploratory construct validation, risk knowledge (19 items), DMT status and actual intention to use DMT were also assessed. Patients participating in PEPADIP piloting completed the PBMS questionnaire twice, i.e. before and after the education program.

We assumed that the predictive power of the PBMS regarding intentions to use DMT would be comparable to results for similar TBP-based approaches in the literature [15-18]. Additionally, exploration of the validity of the PBMS addressed its ability to discriminate sub-groups of patients with different DMT status (currently on / not yet on DMT), on different levels in relation to the educational programme (before / after) and regarding risk knowledge (high / low).

### Validation study

The PBMS was employed in 192 relapsing-remitting MS patients participating in a randomised controlled trial to evaluate the effectiveness of the educational programme PEPADIP [27]. The aim of the program was to enhance critical reflection on scientific evidence and on patients’ own values and preferences and so to enable them to make more informed choices on DMT. Patients completed the PBMS 2 weeks before and 2 weeks after the intervention. As the control group did not receive any specific intervention we considered this subsample appropriate to estimate PBMS re-test-reliability. Besides demographic and general variables, and the patient’s actual intention to start DMT (patient self-administered yes/no reply to a single question), we recorded DMT risk knowledge (patient self-administered 19 item questionnaire) based on an earlier multiple-choice questionnaire [24].

The construct validity of the PBMS was addressed as follows. First, we compared the study groups’ decision making processes on the level of absolute PBMS score values. We assumed the intervention to lead to more critical reflection on DMT (attitude), less willing to comply with the opinions of powerful others (subjective social norm) and greater self-efficacy (perceived control beliefs) compared to the control group. Secondly, based on our assumption that the PBMS-model fit reflects the depth of elaboration of the decision, we compared the study groups regarding the predictive power of the PBMS towards the intention to use DMT. In the same regard we compared patients with high and low risk knowledge and patients already using with those not yet using DMT. Thirdly, as the proportional power of the influence of three PBMS domains on a particular target behaviour might vary [26], we explored the effect of the educational intervention on the relative impact of the three domains. We hypothesized that the influence of the social norm would decrease in favour of control beliefs as a result of the intervention.

### Data analysis

After reversing polarity of the data from negatively poled items, mean scores were calculated for all 6 subscales (range 0–3). PBMS item difficulties between 0.6 and 2.5 (i.e. values within two thirds of the scale range) and standard deviations higher than 0.5 (i.e. half a scale step) were defined as satisfactory. Two levels of risk knowledge (high and low risk knowledge, high, low RK) ) were defined by median split based on the mean DMT risk knowledge score, to produce two groups of comparable size.

According to the expectation-value model, and in particular to the TBP model’s structure, subscale mean values were multiplied pair-wise within each of the three main domains [26]. While the proportion by which the three domains impact on the intention can vary between different behaviours [26], no assumption was available to define specific proportions for the concrete decision-making scenario. An “ intention estimate coefficient” was therefore defined by simply calculating the sum of the three domains’ mean values so that equal weight was given to attitude, subjective social norm and control beliefs (Table 1).

**Table 1:** Effects of educational intervention on PBMS parameters (validation trial)

**PBMS**		**Comparison of PBMS parameters between study groups**			
**Domains**		**control**	**intervention**	**range**	**p**
	**Sub-domains**				
1 Attitude		3.5 (1.9)	3.0 (1.8)	0-9	.04*
	a: expectations regarding outcomes	1.8 (0.6)	1.6 (0.6)	0-3	.001*
	b: values of expected outcomes	1.8 (0.6)	1.8 (0.6)	0-3	.55
2 Social norm		2.1 (1.2)	1.7 (1.0)	0-9	.006*
	a: assumed attitudes of important others	1.9 (0.6)	1.8 (0.7)	0-3	.28
	b: motivation to comply with these norms	1.1 (0.4)	1.0 (0.4)	0-3	.004*
3 Control beliefs		3.2 (2.1)	2.9 (2.1)	0-9	.313
	a: assumed facilitators or barriers	1.6 (0.7)	1.4 (0.8)	0-3	.14
	b: perceived power of these factors	1.8 (0.6)	1.7 (0.7)	0-3	.42
Intention estimate		8.9 (4.1)	7.5 (4.4)	0-27	.04*

P-values refer to unpaired t-tests comparing post intervention mean scores of intervention- and control groups. Theoretical range of scores is 0 to 3 for sub-domains, 0 to 9 for domains and 0 to 27 for intention estimate. “*” indicates statistically significant unpaired t-tests.

Linear regression analyses were conducted according to the model’s structure (Figure 1) which, for the target criterion intention to use DMT (dependent variable), implies three possible sets of predictors (independent variables): (1) the intention estimate (one predictor), (2) the three domains: attitude, subjective social norm and perceived behavioural beliefs, (three predictors) (3) the six sub-domains (six predictors). Simple linear regressions were conducted for each of the 3 predictor sets. R-squares indicated the extent to which intention was explained by the particular predictor set. Comparisons between R-squares of subgroups were conducted using Fisher’s-Z-tests. Pre-post correlations of PBMS scores (at item-, sub-domain and domain-level) within the control group were calculated using Pearson coefficients.

## Results

### Pre-testing

Item difficulties and standard deviations for the PBMS were overall satisfactory (mean 1.6, empiric range 0.24 to 2.4; mean SD 0.92, range 0.92 to 0.5). All but three items showed satisfactory item difficulty and variability of item responses was higher than 0.5 in all items. The PBMS explained 55% of the criterion’s (intention to use or not use DMT) variance when calculated based on the intention estimate (one predictor) and even more, when intention was predicted based on the three domains (60%) or based on the six mean scores of the sub domains (69%) (Table 2).

**Table 2:** Predictive power of PBMS (Pre-test and RCT)

**Sample**	**Measure-ment**	**Outcome**	**PBMS predictor set**		
	**point**		**intention estimate**	**domains**	**sub-domains**
			**1 predictor**	**3 predictors**	**6 predictors**
	pre total n = 50	r^2^	.55(.54)*	.60(.57)*	.69(.65)*
**Pre-test**	pre sub s. n = 19	r^2^	.34(.31)*	.38(.30)	.56*(.42)
	post sub s. n = 19	r^2^	.65(.63)*	.68(.61)*	.73(59)*
	pre total n = 177	r^2^	.41(41)*	.43(41)*	.50(48)*
**RCT**	post total n = 177	r^2^	.61(61)*	.63(62)*	.68(67)*
	post CG n = 88	r^2^	.56(.56)*	.56(.55)*	.64(61)*
	post IG n = 89	r^2^	.69(69)*	.74(73)*	.78(76)*

The table shows R-square(corrected R-square) values (indicating the percentage of explained variance) from predicting the criterion intention to use DMT (disease modifying therapy) using various predictor sets drawn from the PBMS in regression analyses.”*” = significance level <0.01. The pre-test sub sample (n = 19) includes participants of the PEPADIP pilot course who completed the PBMS twice (pre & post). RCT=randomized controlled trial.

Patients not using DMT differed in their responses regarding all but one sub-domain from DMT users whose attitudes and control beliefs were more in favour of DMT (attitude without DMT 2.1, with DMT 4.6, p < .01; control beliefs without DMT 1.6, with DMT 4.7, p < .01) (Table 3). Better risk knowledge was associated with more critical beliefs about possible outcomes of DMT and stronger beliefs in control abilities (domain^1a^: highRK 1.22, lowRK 1.73, p = .009; domain^3a^: highRK: 1.4, lowRK 1.9, p = .016, range 0–3). Regardless of the predictor set (intention estimate, 3 domains or six sub-domains) predictions based on PBMS after the programme were significantly better than before (before 34 to 56%, after 65 to 73% explained variance, p = <.01) (Table 2).

**Table 3:** Exploration of discriminatory validity using DMT status (pre-test) (N = 50)

**PBMS**		**DMT status**				
**Domains**		**pre-test**	**RCT**	**pos-**	**p-value**	
	**Sub-domains**	**without /with**	**without /with**	**sible range**	**pre**	**RCT**
1: Attitude		2.1 / 4.6	2.4 / 4.1	0-9	<.01	<.01
	a: expectations regarding outcomes	1.4 / 2.0	1.5 / 1.9	0-3	<.01	<.01
	b: values of expected outcomes	1.4 / 2.2	1.5 / 2.1	0-3	<.01	<.01
2: Social norm		1.2 / 1.8	1.5 / 2.4	0-9	.23	<.01
	a: assumed attitudes of important others	1.2 / 2.0	1.5 / 2.2	0-3	<.01	<.01
	b: motivation to comply with these norms	1.0 / .9	1.0 / 1.1	0-3	<.01	.34
3: Control beliefs		1.6 / 4.7	2.1 / 4.1	0-9	<.01	<.01
	a: assumed facilitators or barriers	1.0 / 2.1	1.2 / 1.9	0-3	<.01	<.01
	b: perceived power of these factors	1.4 / 2.2	1.5 / 2.0	0-3	<.01	<.01
Intention estimate		4.8 / 11.1	6.0 / 10.1	0-27	<.01	<.01

PBMS results are provided as means separately for groups using or not using DMT (disease modifying treatment), pre-test participants of (N = 50) and participants included in the randomized controlled trial (RCTN = 177). P values refer to unpaired t-tests comparing the two groups.

### Validation study

In the RCT, 192 MS patients were randomly allocated to the control (99) or the intervention group (93). The two groups were comparable with respect to demographic and disease related characteristics (Table 4). Results of PBMS validation are based on 177(88/89) datasets at baseline and 174 (88/86) datasets at follow-up within a 4 week time-frame. All datasets included were complete.

**Table 4:** Demographic data

	**Pre-test sample, n = 50**	**RCT sample, n = 177**			
		**control**	**intervention**	**total**	P*
Female (%)	35 (70)	74 (75)	70 (75)	144 (75)	.93
Age (SD)	41 (9)	37 (10)	36 (11)	36 (11)	.61
Years since diagnosis (SD)	5 (6)	2 (1)	2 (1)	2 (1)	.16
Ongoing DMT (%)	32 (64)	55 (56)	51 (55)	106 (56)	.98

Values are frequencies (percentages) and means (SD). *P values refer to either chi-square tests or to unpaired t-tests. DMT = disease modifying treatment, RCT = randomized controlled trial.

Pre-post correlations within the control group were low on the item level (0.52, range: 0.13 to 0.76) and moderate on the level of sub-domains (0.65, range: 0.57 to 0.73). Contrary to our assumptions, belief composites had changed even in the control group (Table 2). Therefore, interpretation of re-test -reliabilities no longer seemed appropriate.

Regarding absolute PBMS score levels, all single and composed PBMS -parameters of the control group pointed more in the direction of favouring DMT than those of the intervention group. This consistent pattern was statistically significant for the intention estimate (IG 7.5, CG 8.9, p = .04) as well as for attitude and subjective social norm showing that patients in the intervention group held more critical beliefs towards DMT (domain^1^: IG 3.0, CG 3.5, p = .004; domain^1a^: IG 1.8, CG 1.6, p = .001) and were less willing to comply with perceived social norms regarding DMT (domain^2^: IG 1.7, CG 2.1, p = .006; domain^2b^: IG 1.0, CG 1.1, p = .004) (Table 1). The sensitivity of the PBMS questionnaire for actual DMT intentions was reflected in differences regarding PBMS scores between participants using and not yet using DMT. Patients already using DMT were more in favour of DMT in five of six sub-domains (Table 3). Results for the sensitivity of the PBMS to the level of risk knowledge differed slightly from those in pre-tests: No effect was seen regarding control beliefs (domain^3a^: p = .071). However, patients with greater knowledge held more critical beliefs (domain^1a^ 1.8 range 0–3) and were less motivated to comply with assumed norms of important others (domain^2b^: .89; range 0–3) than patients with less knowledge (domain^1a^: 1.5, p. = <.001; domain^2b^:1.2; p = <.001).

Regarding the level of model fit, PBMS explained high percentages of criterion variance for the intention to use DMT. Depending on the predictor set PBMS explained between 41 and 50% at baseline and between 69 and 78% of the variance after educational programme in the total sample (Table 2). All predictor sets showed higher percentages of explained variance in the intervention group (IG) than the control group (CG) (intention estimate: CG 56% / IG 69%, p = .179; three domains CG 56% / IG 74%, p = .047; six sub-domains CG 64% / IG 78%, p = .073) (Table 2).

Using the PBMS to compare the relative influence of TPB domains between the study groups led to ambiguous results. The power to predict attitude was equal in the intervention and control groups. Contrary to our assumption the influence of subjective social norm appeared not to be weakened by the intervention. However, perceived behaviour control turned out to take on a greater relevance in the decision making process after the intervention. In other words, patients’ decisions became more likely to reflect the belief that they had control over the target behaviour and its consequences (Table 5).

**Table 5:** Relative influence of PBMS domains

**N = 177**	**Comparison of the domains’ relative impact**		
	**% explained variance of intention**		**p-value**
	**intervention**	**control**	
**Attitude**	49	45	.76
**Subjective social norm**	31	21	.51
**Perceived behaviour control**	63	50	.25

The table illustrates the predictive power of single domains separately for the study groups. P values for differences between the percentages of explained variance as drawn from regression analyses were calculated using Fishers Z-test.

## Discussion

This study aimed to develop a questionnaire to assess MS patients’ cognitive and emotional representation of decision making processes regarding disease modifying treatments (DMT) based on the theory of planned behaviour (TPB). In pre-testing the questionnaire had already been found to possess convincing item properties and feasibility and encouraging indications of validity. The larger dataset from the RCT yielded further support for the PBMS measurement concept in various regards.

The PBMS was found to possess high predictive power for the target criterion, intention to use DMT. This suggests that the PBMS item pool fully covered the relevant belief composites and that TPB is applicable to this type of decision. PBMS also showed a good sensitivity to differences in beliefs about DMT and the level of corresponding risk knowledge. PBMS response patterns reflected the hypothesised effects of an educational intervention providing factual information about DMT, with responses indicating that the intervention group subsequently showed a more critical attitude and less willingness to comply with social norms. These results correspond with the programme’s underlying aim of enabling patients to make decisions based on personal values and realistic beliefs about expected outcomes in an area where most information is commercially driven. It was expected that the patients’ ability to reflect on these beliefs would be strengthened by providing evidence-based patient information on DMT.

The PBMS showed substantial construct validity by discriminating subgroups with differing DMT status (5 of six domains). Beyond the absolute PBMS score values, the PBMS was also found to be sensitive to the decision support intervention at the level of model fit. The predictive power of the PBMS was higher in the intervention group than in the control group.

This effect is insightful as the basic idea of supporting patients in processing evidence based patient information is to enable patients to reflect on their own priorities when making DMT decisions [28]. Our results showed the intervention group to possess increased cognitive awareness of the belief composites underpinning individual decisions. Cognitive awareness, however, results from deeper elaboration of scientific and internal motivational information relevant to the decision [8]. The latter process complies with evidence based medicine and decision support strategies. These aim to enhance communication and information processing by using decision aids, evidence based patient information tools, educational programmes, and shared decision making. Earlier work by our group showed that, although it may not alter treatment choices, evidence-based patient information can alter decision processes [21]. The results relating to possible shifts in the proportions by which single domains impact on the intention remain ambiguous in the present study. Proportional changes as found in this study could reflect the dynamic character of proportional impact of the TPB domains. However, this dynamic needs further investigation.

The study also has some limitations. First, the selection of TPB out of a pool of more than 30 psychological theories of behaviour change [29] can be challenged. Although evidence for its applicability to various behaviours is substantial, TPB and the rigorous structure of its components (e.g. expectations multiplying values) have been criticized for ascribing too much importance to rational processes [14,30]. As well as rational reasoning, emotional processes and routine behaviour (lacking any decision) also affect motivation when deciding on a specific behaviour [14]. Regardless of the current understanding that emotional processing is essential for and effectively inseparable from cognitive appraisal, we also think that emotional appraisal is adequately represented within each of the sub-domains of our tool (Additional file 1). For instance, by formulating items such as “*The risk I would be taking by putting off immunotherapy for too long frightens me.*” we intended to address emotional aspects of MS-patients’ belief system. On the other hand, the theory’s emphasis on rationality was helpful in focusing on the process of systematic and conscious reflection on the motivation in medical decision making. Our study results confirm our rationale in this regard.

The questionnaire’s additive and multiplicative structure can be challenged for two further reasons. First, the extent of possible hidden redundancy in the items’ underpinning constructs is not yet clear. Second, since our multiple logistic regression analyses were limited to main effects, we cannot exclude interaction effects, e. g “knowledge of risks” may interact with “compliance with norms”. Both would contradict the rigid algorithm to achieve the component scores and the intention / behaviour estimate. However, automatic detection of interactions would have required far greater sample sizes and clear a priori hypotheses for specific interactions. For this initial study we therefore considered it reasonable to adhere to the strategy outlined by Ajzen [9,26]. The conversion of an intention into action is determined not only by the person’s intention but also by personal and environmental barriers and by the person’s volitional control. In this regard, our results have to be seen as preliminary. However, we are awaiting data on DMT uptake from the PEPADIP trial which will enable us to validate the PBMS using the actual target behaviour rather than a substitute. It can be argued that some of our results could emerge by chance due to multiple testing. Indeed, the study was not sufficiently powered to keep all reported differences when using an alpha level adjusted to a number of six tests conducted in parallel. However, our testing was driven by clear hypotheses referring to the complete model. Moreover, as these hypotheses refer to a given fixed hierarchical structure, multiplicity of the used parameters is limited. Similarity of the results of pre-test and controlled trial provides further support for their relevance. Finally, it was not possible to employ a test-retest strategy to estimate PBMS reliability because the control group showed considerable change in some of the domains between pre- and post-intervention. However, three and six months follow up data are now available for the PBMS. Here, no further change was detected, either for the control group or for the total sample, and test-retest reliabilities were satisfactory for all six domains (.74; .71; .79, 79; .60; .71) based on 155 data sets. Further studies will be necessary to replicate these results.

Modeling behavioural decisions in terms of TPB conforms to basic assumptions of patient empowerment, since it takes into account the social situation (subjective social norm) and self-efficacy (control beliefs) as well as attitude. Substantial evidence has shown the TPB to be helpful for developing interventions motivating change of specific health behaviours such as condom use, exercise, diet, and medication adherence [11,18]. Our study is in agreement with a number of other studies that have used TPB to elucidate inherent processes in individuals making health related decisions [12-14,30]. When developing and evaluating the effectiveness of theory based interventions, it is useful to be able to identify specific underlying beliefs and measure their impact on informed decision making. This is particularly important, as better decisions, in terms of shared decision making or the informed choice cannot (by definition) result in immediate health improvement. Our study shows how theory can be used to inform the design of an effective intervention and to guide its evaluation. However, since the TPB itself is generic and its domains are quite elementary, we feel that it has great potential for developing specific applications for other decision support contexts as well. This would answer the call for more theoretical foundation for decision support strategies [31,32]. Using the tool will help us to further develop end-points for evaluation of patient empowerment and shared-decision making, for example by making measures such as the Multimodal Measure of Informed Choice more specific [3].

## Conclusions

The study yielded first indicators for the validity of the PBMS as a tool to provide fine grained analysis of inherent processes in patients when making choices about DMT. The questionnaire was sensitive to patients’ change in attitude and motivation to comply with social norms evoked by an educational intervention. Moreover, as reflected in the overall model fit, the questionnaire also appeared to be sensitive to varying levels of depth of decision elaboration. The use of the TPB as shown in the development of the PBMS questionnaire is easily applicable to other decision making scenarios.

## Competing interests

CH has received grants from Biogen-Idec, Merck-Serono, Novartis Pharma and Teva Pharma as well as speaker’s fees. AS has received a board membership fee from Novartis and speaker’s fees from Sanofi-Aventis. SK is supported by a rehab-fellowship grant from the National MS Society, USA. JK and KF have received travel expenses from Merck Serono. IB has no conflict of interest.

## Authors’ contributions

Conceived and designed the experiments: JK, SK, CH, KF,NS. Performed the experiments: SK, KF, IB. Analysed the data: JK. Contributed reagents/materials/analysis tools: SK, IB, KF, NS,AS. Wrote the manuscript: JK, SK, CH, AS. All authors read and approved the final manuscript.

## Pre-publication history

The pre-publication history for this paper can be accessed here:

http://www.biomedcentral.com/1472-6947/12/60/prepub

## Supplementary Material

Additional file 1 **Appendix 1.** English translation of the PBMS questionnaire, 30 items as used in the PEPADIP trial. Domains and sub-domains are (in this table but not in the original questionnaire) indicated using symbols (❶=domain^1a^: expectations regarding outcomes; ①= domain^1b^: values of outcomes; ❷= domain^2a^: subjective social norm; ②= domain^2b^: motivation to comply; ❸= domain^3a^: expectations regarding control; ③= domain^3b^: value of control factors and perceived power). Click here for file
