# Supplementary material for: Applying the theory of planned behaviour to multiple sclerosis patients’ decisions on disease modifying therapy – questionnaire concept and validation
Source: BMC Med Inform Decis Mak. 2012 Jul 2;12:60. doi: 10.1186/1472-6947-12-60 (PMC3416666; doi:10.1186/1472-6947-12-60)
Supplement: Additional file 1 — Appendix 1. English translation of the PBMS questionnaire, 30 items as used in the PEPADIP trial. Domains and sub-domains are (in this table but not in the original questionnaire) indicated using symbols (❶=domain1a: expectations regarding outcomes; ①= domain1b: values of outcomes; ❷= domain2a: subjective social norm; ②= domain2b: motivation to comply; ❸= domain3a: expectations regarding control; ③= domain3b: value of control factors and perceived power). [file 1472-6947-12-60-S1.doc]

Appendix 1

| **PBMS**  This questionnaire assesses your position on immunotherapy for your MS. The following statements express different attitudes on immunotherapy: | | | | | |
| --- | --- | --- | --- | --- | --- |
| **Please tick the box that best describes your personal agreement with each statement. Please do not miss any statement out** | | **I disagree** | **I somewhat disagree** | **I somewhat agree** | **I agree** |
| 1 | Immunotherapy can have a positive effect on my disease course. ➊ | 🞎 | 🞎 | 🞎 | 🞎 |
| 2 | Most people in my situation would use immunotherapy. ➋ | 🞎 | 🞎 | 🞎 | 🞎 |
| 3 | I am not doing badly enough for immunotherapy. ➌ | 🞎 | 🞎 | 🞎 | 🞎 |
| 4 | The risk I would be taking by putting off immunotherapy for too long frightens me.➀ | 🞎 | 🞎 | 🞎 | 🞎 |
| 5 | I find it difficult to discuss immunotherapy with my doctor.➁ | 🞎 | 🞎 | 🞎 | 🞎 |
| 6 | I can hardly imagine going through with a therapy when it is uncertain whether I will benefit from it at all. ➂ | 🞎 | 🞎 | 🞎 | 🞎 |
| 7 | The only help for MS is immunotherapy. ➊ | 🞎 | 🞎 | 🞎 | 🞎 |
| 8 | People who are close to me expect me to do without immunotherapy. ➋ | 🞎 | 🞎 | 🞎 | 🞎 |
| 9 | The future course of my MS will ultimately also depend on other factors in addition to immunotherapy. ➌ | 🞎 | 🞎 | 🞎 | 🞎 |
| 10 | I don’t believe I need therapy at all. ➀ | 🞎 | 🞎 | 🞎 | 🞎 |
| 11 | My doctor knows best whether or not immunotherapy is right for me. ➁ | 🞎 | 🞎 | 🞎 | 🞎 |
| 12 | For me, it’s easier to do something rather than to wait and see. ➂ | 🞎 | 🞎 | 🞎 | 🞎 |
| 13 | Many people benefit from immunotherapy. ➊ | 🞎 | 🞎 | 🞎 | 🞎 |
| 14 | My doctor says that immunotherapy can be of benefit to me. ➋ | 🞎 | 🞎 | 🞎 | 🞎 |
| 15 | I expect immunotherapy will be effective in my case. ➌ | 🞎 | 🞎 | 🞎 | 🞎 |
| 16 | It is important for me not to reject a chance for a possible benefit. ➀ | 🞎 | 🞎 | 🞎 | 🞎 |
| 17 | It’s difficult for me to go against the advice of my friends and family. ➁ | 🞎 | 🞎 | 🞎 | 🞎 |
| 18 | I trust myself to go through with immunotherapy and everything it involves. ➂ | 🞎 | 🞎 | 🞎 | 🞎 |
| 19 | The evidence for the effectiveness of immunotherapy is not convincing. ➊ | 🞎 | 🞎 | 🞎 | 🞎 |
| 20 | My family would like me to have immunotherapy. ➋ | 🞎 | 🞎 | 🞎 | 🞎 |
| 21 | It could be that I use immunotherapy for years without actually benefiting from it.➌ | 🞎 | 🞎 | 🞎 | 🞎 |
| 22 | An immunotherapy that only reduces relapses but does not influence the disease course would not be important for me. ➀ | 🞎 | 🞎 | 🞎 | 🞎 |
| 23 | The opinions of other people who have looked into the topic of immunotherapy are not important to me. ➁ | 🞎 | 🞎 | 🞎 | 🞎 |
| 24 | The idea of what immunotherapy might do to my body frightens me.➂ | 🞎 | 🞎 | 🞎 | 🞎 |
| 25 | A lot of people who have immunotherapy experience unpleasant side-effects. ➊ | 🞎 | 🞎 | 🞎 | 🞎 |
| 26 | In my current situation side-effects would have a very negative impact on my everyday life. ➀ | 🞎 | 🞎 | 🞎 | 🞎 |
| 27 | I can easily shake off any pressure other people put on me. ➁ | 🞎 | 🞎 | 🞎 | 🞎 |
| 28 | Injections are not a problem for me. ➀ | 🞎 | 🞎 | 🞎 | 🞎 |
| 29 | It’s up to me alone whether I have immunotherapy or not. ➁ | 🞎 | 🞎 | 🞎 | 🞎 |
| 30 | I am frightened by the risks associated with immunotherapy. ➀ | 🞎 | 🞎 | 🞎 | 🞎 |

English translation of the PBMS questionnaire, 30 items as used in the PEPADIP trial. Domains and sub-domains are (in this table but not in the original questionnaire) indicated using symbols (➊=domain^1a^: expectations regarding outcomes; ➀= domain^1b^: values of outcomes; ➋= domain^2a^: subjective social norm; ➁= domain^2b^: motivation to comply; ➌= domain^3a^: expectations regarding control; ➂= domain^3b^: value of control factors and perceived power).
